# Propofol Total Intravenous Anesthesia for Pediatric Proton Radiotherapy and Its Effect on Patient Outcomes

**DOI:** 10.3390/cancers17121904

**Published:** 2025-06-07

**Authors:** Pascal Owusu-Agyemang, Julie Mani, Techecia Idowu, Acsa Zavala, January Tsai, Ravish Kapoor, Olakunle Idowu, Jose Galdamez Melara, Pallavi Muraleedharan, Clara Francis, Lei Feng, Juan Cata

**Affiliations:** 1 Department of Anesthesiology and Perioperative Medicine, The University of Texas MD Anderson Cancer Center, Houston, TX 77030, USAjcata@mdanderson.org (J.C.); 2 Anesthesiology and Surgical Oncology Research Group, Houston, TX 77030, USA; 3 The University of Texas Health Science Center at Houston, Houston, TX 77030, USA; jose.m.galdamezmelara@uth.tmc.edu (J.G.M.); pallavi.muraleedharan@uth.tmc.edu (P.M.); 4 College of Osteopathic Medicine, Sam Houston State University, Huntsville, TX 77241, USA; 5 Department of Biostatistics, The University of Texas MD Anderson Cancer Center, Houston, TX 77030, USA

**Keywords:** pediatric, anesthesia, cancer, outcomes

## Abstract

**Background:** Patient motion poses significant challenges for the accurate delivery of radiotherapy. In children undergoing proton beam therapy (PBT), up to 30 treatments under general anesthesia may be required over a period of 6 to 8 weeks. To date, the impact of this many iterative anesthetic exposures on patient outcomes remains unclear. **Objective:** The primary objective of this study was to assess the impact of iterative anesthesia with propofol-based total intravenous anesthesia (propofol-TIVA) on overall survival. The secondary objective was to assess the association between propofol-TIVA and the occurrence of an unplanned admission or emergency room visit within 30 days of treatment start. **Methods:** This was a retrospective study of children (≤19 years) who had undergone PBT (with or without anesthesia) for central nervous system disease. The Log-rank test and Cox proportional hazards models were used for analysis. Propensity score matching and E-value analyses were used to adjust for selection bias. **Results:** The average age of the 461 children included was 9.0 years (SD ± 4.9). The majority, 261/461 (56.6%), were male, and 267/461 (57.9%) had undergone PBT without anesthesia. The group who underwent PBT with propofol-TIVA were younger (4.7 years vs. 12.2 years, *p* < 0.001) and had higher proportions of patients with treatment interruptions (111/194 [57.2%] vs. 118/267 [44.2%], *p* = 0.006), chemotherapy history (64/194 [33.0%] vs. 18/267 [6.7%], *p* < 0.001), concurrent chemotherapy (37/194 [19.1%] vs. 27/267 [10.1%], *p* = 0.006), and unplanned admissions/emergency room visits (26/194 [13.4%] vs. 1/267 [0.4%], *p* < 0.001). Overall survival rates (propofol-TIVA vs. no anesthesia) at 1yr (94% vs. 96%), 2 years (88% vs. 90%), and 3 years (88% vs. 89%) were similar between patient groups (*p* = 0.558). In the multivariable analysis, PBT with propofol-TIVA was associated with increased odds of an unplanned admission/emergency room visit before (OR, 38.311; 95%CI, 5.139–285.580; *p* < 0.001) and after (OR, 42.012; 95% CI, 5.322–331.632; *p* < 0.001; E-value = 83.52) propensity score matching. **Conclusions:** In this retrospective study of children undergoing PBT for central nervous system disease, there was no association between anesthesia exposure with propofol-based total intravenous anesthesia and overall survival. However, PBT with propofol-based total intravenous anesthesia was associated with an increased risk of an unplanned admission/emergency room visit within 30 days of treatment start.

## Simple Summary

To facilitate the safe and accurate delivery of radiotherapy, children undergoing proton beam therapy may require multiple anesthetic exposures over a period of 6 to 8 weeks. Research suggests the choice of anesthetic agent may influence cancer progression. To date, the influence of this many anesthetic exposures on the short- and long-term outcomes of children undergoing radiotherapy is unknown. In this retrospective study, multiple anesthetic exposures with propofol-based total intravenous anesthesia did not influence the survival of children undergoing proton beam therapy. However, compared to children who completed proton beam therapy without anesthesia, those who were undergoing treatment with propofol-based anesthesia were 38 times more likely to require an unplanned admission or emergency room visit. Further studies involving the use of other anesthetic agents may guide practitioners in selecting the preferred agents for this group of patients.

## 1. Introduction

Preclinical studies suggest the choice of anesthetics may impact oncologic outcomes [1,2]. Among these studies, the effects of volatile anesthetics and opioids have often been described as pro-metastatic, whereas propofol is mostly described as anti-oncogenic and anti-metastatic. However, the results of large randomized clinical trials on this subject have not shown any relevant differences in cancer-specific survival, making the clinical impact of the preclinical data unclear [3,4,5,6,7,8].

There are several probable explanations as to why the preclinical findings have not consistently translated into clinical results [1]. An important example is the very high doses of anesthetics that are necessary for any significant oncogenic or anti-oncogenic effects to be observed during in vitro studies [1,2]. For example, the anti-oncogenic effects of propofol have been consistently demonstrated after long periods of exposure (more than 24 h), with the most significant effects observed at doses of 10 µg/mL or higher, doses that are much higher than those used clinically [1].

It is also becoming increasingly evident that cancer-specific outcomes after exposure to volatile anesthetics or propofol may be comparable [9]. However, due to clinical limitations, few studies have been able to assess whether the oncologic outcomes of patients exposed to anesthetics differ from those of patients who did not require anesthesia for similar procedures [10,11]. Taken together, these limitations significantly impact the interpretation of available studies on anesthesia and cancer outcomes. Additional insight may be gained by studying oncologic outcomes after procedures where patients may or not require multiple general anesthetic exposures over a period of time. Hypothetically, exposure to multiple anesthetics over a period of time may serve as a surrogate for prolonged periods of exposure, and comparisons to the outcomes of patients who are able to complete their treatments without anesthesia may provide added information into assessing whether exposure to anesthetics does indeed impact oncologic outcomes.

Patient immobility is critical to ensuring the accuracy and safety of proton beam therapy. In adults and older children, this is normally achieved without anesthesia or sedation. However, attaining this degree of immobility and compliance in younger children is difficult. As a result, some children undergoing proton beam radiotherapy (PBT) may require up to 30 treatments under general anesthesia over a period of 6 to 8 weeks, with each of these treatments lasting up to 60 min [12]. This patient population provides an opportunity to assess the oncologic impact of repeated exposures to anesthetics and to compare the outcomes of children who complete their treatments with the aid of general anesthesia to those who complete them without anesthesia. In addition, it also provides an opportunity to assess the impact of multiple anesthetic exposures on the short-term outcomes of children undergoing PBT.

To this end, we conducted a single-institution retrospective study with the primary objective of comparing the survivals of children who had undergone PBT for central nervous system (CNS) malignancies under propofol-based total intravenous anesthesia (TIVA), which is the standard technique at our institution, to those of children who completed their PBT for CNS malignancies without anesthesia. Our secondary objective was to assess whether there was any association between undergoing PBT under propofol-based TIVA and the occurrence of an unplanned admission or emergency room (ER) visit within 30 days of the start of treatment. Based on the available preclinical data, our hypothesis was that children undergoing PBT under propofol TIVA would benefit from improved accuracy of radiotherapy and the potential anti-oncogenic effects of repeated infusions of propofol and thus achieve oncologic outcomes that are superior to those of children who completed their treatments without anesthesia [13,14].

## 2. Methods

This retrospective study was approved by the institutional review board (IRB) of The University of Texas MD Anderson Cancer Center (IRB # PA 16-0160). The requirement for written informed consent was waived by the IRB, and the findings are reported according to the Strengthening the Reporting of Observational Studies in Epidemiology guidelines.

### 2.1. Study Population

**Figure 1:**
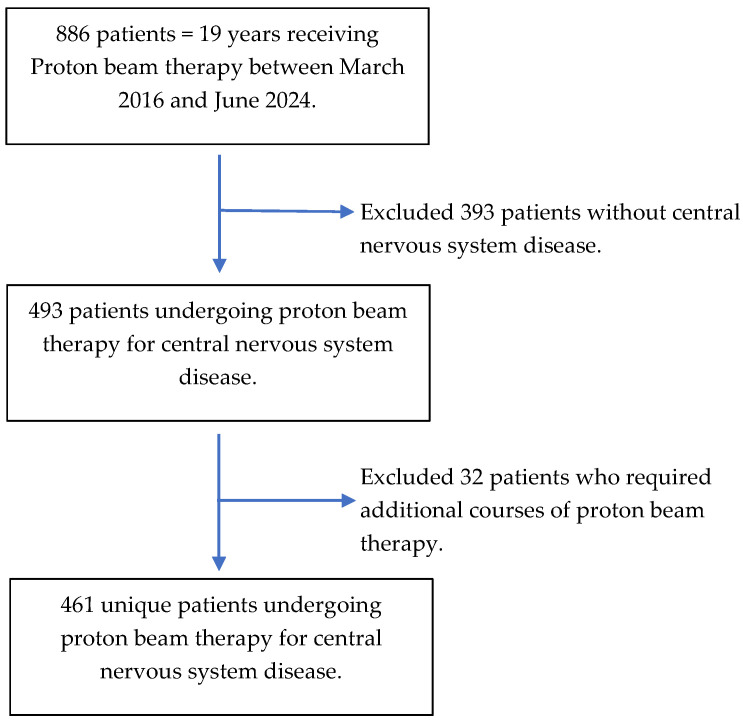
Patient selection process.

Children (≤19 years of age) who had undergone PBT for central nervous system (CNS) malignancy at our comprehensive cancer center between March 2016 and June 2024 (N = 493) were included in this study. Our comprehensive cancer center is devoted exclusively to cancer patient care, research, education, and prevention. To limit the potential confounding influence of recurrent disease, only the index-prescribed course of PBT visits was considered for analysis (N = 461). The patient selection process is illustrated in Figure 1.

### 2.2. Study Covariates

Study covariates were extracted from subsections of the institutional electronic data warehouse and stored on the Research Electronic Data Capture web platform hosted by the University of Texas MD Anderson Cancer Center. Patient demographics, including age, gender, race, and ethnicity, were recorded. In addition, patient diagnoses and the potentially confounding oncologic variables of a prior history of chemotherapy, number of chemotherapy agents used, concurrent chemotherapy, PBT timing (date of PBT minus date of tumor surgery), PBT field of treatment (brain, spine, or craniospinal), interruptions to the prescribed course of PBT (number of days), and the reasons for interruption were recorded. The reasons for treatment interruption were classified as medical, non-medical, or unknown (when no reason was recorded). The endpoints necessary for calculating overall survival (OS) including the start date of PBT, current status (alive or deceased), and date of last follow-up alive, or date of death, were also recorded.

Regarding the secondary objectives of the study, any inpatient admission or emergency room visit within thirty days of the start of PBT, as well as the symptoms and diagnosis reported at admission, were recorded.

### 2.3. Anesthetic Management and Exposure Variable

At our institution, the current practice for managing patients who require anesthesia for PBT is propofol-based TIVA with spontaneous ventilation. Unless contraindicated, ondansetron is routinely administered for the prophylaxis of postoperative nausea and vomiting, and dexmedetomidine boluses are administered to children who exhibit severe emergence delirium. Children who choose to undergo PBT without anesthesia do not receive any sedatives or anesthetics from the medical team.

The exposure variable of interest was propofol-based TIVA, and patients were divided into two groups: those who completed PBT with the aid of propofol-based TIVA (propofol anesthesia group) and those who completed their treatments without anesthesia (no anesthesia group).

### 2.4. Statistical Analysis

Patients’ demographics, treatment, and clinical outcomes were summarized through descriptive statistics. Wilcoxon rank sum test was used to compare location parameters of continuous distributions between patient groups. The chi-square test or Fisher’s exact test was used to evaluate the association between two categorical variables. Overall survival was calculated from the start of PBT to the date of death. Patients who were alive at the time of this study were censored at the last follow-up date. The Kaplan–Meier method was used to estimate OS. Median OS time in months with 95% confidence interval was calculated. The Log-rank test was used to evaluate the difference in OS between patient groups. Cox proportional hazards models were used for multivariable analysis. Collinearity diagnostics were performed and indicated no collinearity problem. The Schoenfeld residual was used to check the proportional hazards assumption.

A multivariate logistic regression model was fitted to evaluate the association between study covariates and the status of an unplanned admission or ER visit. The full model included the covariates with *p*-value < 0.10 from the univariate analysis, and a backward selection method was used. The final model (number of events/number of patients [E/N] = 27/461) included the covariates with *p*-value less than 0.15.

To adjust for selection bias, we conducted a propensity score matching analysis of unplanned admissions/ER visits. The propensity score is the conditional probability of status (patients who underwent PBT with propofol anesthesia) conditional on a set of observed covariates. In this analysis, we included the following prognostic covariates in the multicovariable logistic model to estimate the propensity scores: chemotherapy history and the field of PBT. These covariates were significantly imbalanced between the group of patients who had propofol anesthesia and the group of patients who did not have anesthesia (*p* value < 0.0001, 0.0003).

The Greedy 5 → 1 digit match algorithm was used to match the baseline covariates, so that the two groups (propofol anesthesia vs. no anesthesia) would have similar propensity scores. A total of 148 patients who had propofol anesthesia and had non-missing values for the covariates were matched on a 1:1 ratio to 148 patients who did not have anesthesia and had non-missing values for the covariates. As shown in Supplemental Table S1, the standardized differences for all covariates were <0.1% in the post-matching cohort, suggesting a substantial reduction in bias between the two groups. To evaluate how strong a relationship between a confounder not represented in the analysis would need to be to result in a disruption of the observed effect of TIVA on unplanned admissions, an E-value analysis was performed. Statistical software SAS 9.4 (SAS, Cary, NC, USA) and S-Plus 8.2 (TIBCO Software Inc., Palo Alto, CA, USA) were used for all the analyses.

## 3. Results

### 3.1. Baseline Characteristics

A total of 461 children were included in this study. Their median age (Interquartile range [IQR]) was 9 years [IQR, 5–13] and 261/461 (56.6%) were male. The majority (267/461 [57.9%]) completed their course of PBT without anesthesia (no anesthesia group) and 194/461 (42.1%) completed their treatments with the aid of propofol-TIVA (propofol anesthesia group). Compared to the no anesthesia group, the propofol anesthesia group was younger (median age [IQR]; 4 years [IQR, 3–6] versus 12 years [10,11,12,13,14,15]; *p* < 0.001), and had higher proportions of children who had received prior chemotherapy (64/194 [33.0%] versus 18/267 [6.7%]; *p* < 0.001) or concurrent chemotherapy (37/194 [19.1%] versus 27/267 [10.1%]; *p* = 0.006). The propofol anesthesia group also had a significantly higher proportion of patients who had/or were receiving three or more chemotherapy agents (38/194 [19.6%] versus 12/267 [4.5%] in the no anesthesia group; *p* < 0.001) and a higher proportion of patients undergoing craniospinal radiation (55.7% versus 37.1%, *p* < 0.001). Interruptions to the prescribed course of PBT (Yes/No) were significantly higher in the propofol anesthesia group (111/194 [57.2%] versus 118/267 [44.2%]; *p* = 0.006), with a higher proportion of treatments in the propofol anesthesia group interrupted due to equipment failure or non-medical reasons (50/194 [25.8%] versus 34/267 [12.7%]; *p* < 0.001). Furthermore, the number of interruptions to the course of PBT was also significantly higher among the propofol anesthesia group, with 68/194 (35.1%) having more than one interruption, compared to 68/267 (25.5%) in the no anesthesia group, *p* = 0.019. Other baseline and clinical characteristics of the study population are shown in Table 1.

### 3.2. Oncological Outcome

The median follow-up time was 22.2 months (95% CI: 18.5~28.6 months). The median OS for the entire study population had not been reached. Beyond the median follow-up time of 22.2 months, there were 198 and 157 patients at risk at 24 and 36 months, respectively. The OS rates at 1, 2, and 3 years along with the 95% confidence intervals for all patients, as well as for each of the subgroups studied, are presented in Table 2.

**Figure 2:**
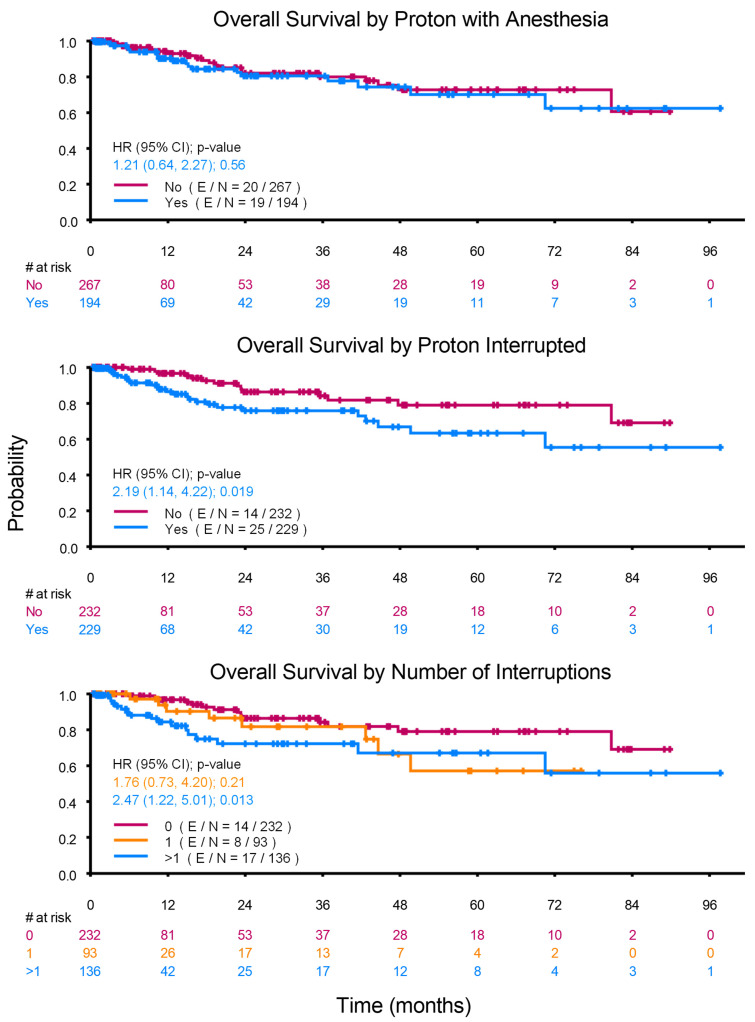
Kaplan-Meier Plots. Undergoing radiotherapy with propofol anesthesia did not impact survival. Interruptions to the course of radiotherapy and a higher number of days of interruption were each associated with worse survival.

As shown in Figure 2, the OS of the propofol anesthesia and no anesthesia groups were not significantly different (*p* = 0.558). Furthermore, the univariate Cox proportional hazards model did not demonstrate an association between age (as a continuous variable) and OS (Hazard Ratio, 0.961; 95% CI, 0.901–1.025; *p* = 0.227). Of the variables included, interruptions to the prescribed course of PBT (*p* = 0.019), a higher number of PBT interruptions (*p* = 0.033), and concurrent chemotherapy (*p* = 0.003) were each associated with worse survival.

### 3.3. Thirty-Day Unplanned Admissions or Emergency Room Visits

Of the 461 children included in this study, 27 (5.9%) had an unplanned admission or ER visit within 30 days of the start of PBT. Before propensity score matching (Table 3), the group of children who had an unplanned admission or ER visit was younger (median age [IQR], 4 years [2,3,4,5,6,7,8] versus 9 years [5,6,7,8,9,10,11,12,13]; *p* < 0.001) and had a higher proportion of children who had received propofol anesthesia (26/194 [13.4%] versus 1/267 [0.4%]; *p* < 0.001). In addition, higher proportions of children who had received previous chemotherapy (9/82 [11.0%] versus 18/379 [4.7%]; *p* = 0.029) and those who had interruptions to their prescribed course of PBT (19/229 [8.3%] versus 8/232 [3.4%]; *p* = 0.027) had an unexpected admission or ER visit. After propensity score matching (Table 3), a greater proportion of children who received propofol-TIVA required an unplanned admission/ER visit (17/148 [11.5%] versus 1/147 [0.7%] in the no anesthesia group; *p* < 0.001). The symptoms at readmission and the diagnoses at readmission are shown in Supplemental Tables S2 and S3, respectively.

In the multivariable analysis before propensity score matching, children who had undergone PBT with propofol-TIVA had significantly higher odds of requiring an unplanned admission or ER visit than those who completed their treatments without anesthesia (OR, 38.311; 95% CI, 5.139–285.580; *p* < 0.001). Interruptions to the course of PBT were not associated with an unexpected 30-day admission or ER visit (OR, 1.968; 95% CI, 0.819–4.727; *p* = 0.1300). After propensity score matching, PBT with propofol-TIVA (OR, 42.012; 95% CI, 5.322–331.632; *p* < 0.001; E-value = 83.52) and age (OR, 1.166; 95 CI, 1.003–1.357; *p* = 0.0461) were each associated with higher odds of an unplanned admission/ER visit. The observed E-value of 83.52 implies that considerable unmeasured confounding would be needed to explain away the effect of propofol-TIVA on unplanned admissions.

## 4. Discussion

In this retrospective study of 461 children who had undergone PBT for CNS disease, there was no significant difference in overall survival when comparing children who received propofol-based TIVA to those who underwent treatment without anesthesia. However, undergoing PBT under propofol-based TIVA was associated with more than a 38-fold increase in the odds of an unplanned admission or ER visit within thirty days of the start of PBT.

To date, there is limited data on the direct effect of propofol on tumors of CNS origin. The few available studies have focused on glioma and glioblastoma cells and have produced mixed results [14,15,16,17,18]. For example, in two separate in vitro studies, propofol was shown to suppress glioma cell proliferation and invasion via modulation of circular and micro-RNA expression [14,15]. However, in another in vitro study on glioma cells, Cen and colleagues showed that compared to sevoflurane, propofol significantly downregulated the potential tumor suppressors SERPINI1 and CAMK2A and upregulated the important immune checkpoint molecule CD274 (PDL 1), potentially resulting in the suppression of immune cell function and immune escape of glioma cells [16].

Furthermore, the results of the available retrospective studies suggest that the type of general anesthetic does not influence the survival of patients undergoing glioma or glioblastoma resection [17,18]. It remains unclear whether the contradictory results of the available in vitro studies have any influence on these findings. However, an often-cited limitation of retrospective studies has been the comparatively shorter duration of anesthetic exposure and the lower dose of anesthetics that are used in clinical settings. Regarding how these limitations relate to our study, it is plausible that although propofol infusions were administered on multiple consecutive days over a period of 6 to 8 weeks, the plasma levels attained in our patient population may not have been sufficient to observe any beneficial effect. It is also plausible that the pro- and anti-oncogenic effects of propofol may be effective concurrently, resulting in no influence on the survival of children undergoing radiotherapy for CNS disease. Lastly, the effect of propofol on tumors which have been exposed to multiple doses of radiotherapy and the anti-tumor effect of prolonged propofol exposure is unknown.

Similar to our findings, previous studies in adult patients have reported worse survival in patients who experienced interruptions to their course of radiotherapy [19,20]. For example, in a retrospective study of 35,845 patients with breast cancer, an increase in the number of days of interruption was associated with a corresponding increase in the likelihood of mortality [19]. Significantly, interruptions of as little as 2 days significantly impacted OS. In our study, 2 or more days of treatment interruption was associated with up to 15% decline in survival rates. It is notable that there was a significantly higher number of treatment interruptions in the propofol anesthesia group, with a significantly higher proportion related to malfunction of radiotherapy equipment or non-medical reasons. Cancelations of anesthesia cases on days when there was malfunction of radiotherapy equipment may have been related to scheduling constraints. Cancelations for non-medical reasons may have been related to anesthesia-related concerns such as violation of fasting guidelines.

To date, there is limited data on the rates of unexpected readmissions after pediatric radiotherapy. In a retrospective review of 1116 consecutive patients, the unexpected readmission rate among the 14 children included was similar to ours (5%) [21]. However, there were no comparisons between children who had received anesthesia for their treatments and those who had not. In our study, the reasons for the observed higher rates of readmission among children receiving anesthesia are not entirely clear. However, readmissions for fever and infectious complications were confined to the propofol anesthesia group. This may suggest that patients in the propofol anesthesia group were more prone to infection either due to their younger age or higher likelihood of prior and concurrent chemotherapy. In addition, propofol has been shown to suppress T-cell function, inhibit macrophage phagocytosis, and increase host susceptibility to microbial infection [22,23,24]. Thus, it is probable that our observation may suggest added immunosuppression from repeated exposure to propofol in the sub-group of children who received propofol-based TIVA for their treatments. It is also probable that frequent access of central venous lines and salient aspirations in the anesthesia group may have contributed to a higher incidence of infectious and pulmonary complications.

Regarding anesthetic technique for pediatric radiotherapy, there is currently limited data to suggest the superiority of propofol TIVA over anesthesia with volatile agents or vice versa. In a retrospective study of 739 pediatric external beam radiation procedures, anesthesia was maintained with sevoflurane and a laryngeal mask airway. The reported rate of complications was 15.7% and included nausea, vomiting, hypotension, laryngospasm, and aspiration [25]. Similar to our practice, other studies have described the use of propofol-TIVA with spontaneous ventilation and supplemental oxygen by nasal canula and reported lower complication rates (1.3%) [26]. However, further research is required to make definitive conclusions about the preferred anesthetic technique. It is notable that the risk of complications during propofol-TIVA increases with higher propofol dose, increased procedure duration, and the use of adjuncts such as benzodiazepines, opioids and ketamine [26].

There are several limitations to our study. Firstly, its retrospective nature and small sample size significantly limits the interpretation of our findings, especially those pertaining to the survival analysis. Secondly, serum propofol concentrations were not measured and it is unclear whether the concentrations attained were similar to those used during in vitro studies. Moreover, our study included children diagnosed with several tumor types, the anesthetic effects on many of which have not been studied. It is thus unclear whether the available preclinical and clinical data make for appropriate references or hypothesis creation. Lastly, our cancer center is located in an urban area with two other major children’s hospitals and their affiliate clinics. Thus, unexpected readmissions or ER visits to institutions outside our hospital network may not have been captured and could have resulted in an underestimation of unexpected readmissions.

## 5. Conclusions

In conclusion, in this single-institution study of children undergoing PBT, undergoing treatments with the aid of propofol-based TIVA did not impact survival but significantly increased the odds of an unexpected admission within thirty days of the start of treatment. The single-institution nature of our study and the small sample size limit the generalizability of these findings.

## Figures and Tables

**Table 1:** Demographic and clinical characteristics of 461 children who underwent proton beam therapy with or without propofol anesthesia.

Variable	All Patients (*n* = 461)	No Anesthesia (*n* = 267)	Propofol Anesthesia (*n* = 194)	*p*-Value
Age, years, median	9	13	4	<0.001
(interquartile range)	(5–13)	(10–16)	(2–6)	
Gender, No. (%)				0.975
Female	200 (43.4)	116 (43.4)	84 (43.3)	
Male	261 (56.6)	151 (56.6)	110 (56.7)	
Ethnicity/Race of patients with available data ^a^, No. (%)				0.520
American Indian or Alaska Native	2 (0.5)	0 (0)	2 (1.1)	
Asian	31 (7.3)	19 (7.7)	12 (6.9)	
Black or African American	46 (10.9)	29 (11.7)	17 (9.7)	
Hispanic or Latino	182 (43)	103 (41.5)	79 (45.1)	
White	162 (38.3)	97 (39.1)	65 (37.1)	
Prior Chemotherapy, No. (%)				<0.001
No	379 (82.2)	249 (93.3)	130 (67)	
Yes	82 (17.8)	18 (6.7)	64 (33)	
Concurrent Chemotherapy, No. (%)				0.006
No	397 (86.1)	240 (89.9)	157 (80.9)	
Yes	64 (13.9)	27 (10.1)	37 (19.1)	
Number of Chemotherapy Agents, No. (%)				<0.001
None	346 (75.1)	230 (86.1)	116 (59.8)	
One	31 (6.7)	11 (4.1)	20 (10.3)	
Two	34 (7.4)	14 (5.2)	20 (10.3)	
Three or more	50 (10.8)	12 (4.5)	38 (19.6)	
Days between Cancer-Related Surgery and Start of PBT, mean (SD)	241 (569)	281 (663)	186 (406)	0.898
Proton Radiotherapy Field, No. (%)				<0.001
Brain	234 (50.8)	156 (58.4)	78 (40.2)	
Craniospinal	207 (44.9)	99 (37.1)	108 (55.7)	
Spine	20 (4.3)	12 (4.5)	8 (4.1)	
PBT Interrupted, No. (%)				0.006
No	232 (50.3)	149 (55.8)	83 (42.8)	
Yes	229 (49.7)	118 (44.2)	111 (57.2)	
Days of Interruption, No. (%)				0.019
1	93 (20.2)	50 (18.7)	43 (22.2)	
≥2	136 (29.5)	68 (25.5)	68 (35.1)	
Reasons for Interruption, No. (%)				
Medical	123 (26.7)	66 (24.7)	57 (29.4)	0.264
Equipment Failure and Non-medical	84 (18.2)	34 (12.7)	50 (25.8)	<0.001
Unknown	62 (13.4)	31 (11.6)	31 (16)	0.175
30-day Admission and/or ER Visit, No. (%)				<0.001
No	434 (94.1)	266 (99.6)	168 (86.6)	
Yes	27 (5.9)	1 (0.4)	26 (13.4)	

Abbreviations: SD, standard deviation; PBT, proton beam therapy: ER, emergency room. ^a^ Number of children with unknown race/ethnicity = 38.

**Table 2:** The overall survival rates at one, two, and three years after initiation of proton beam therapy.

Variable	Patients	Events	OS Rate at 1 Year	OS Rate at 2 Years	OS Rate at 3 Years	*p*-Value *
			(95%CI)	(95%CI)	(95%CI)	
All patients	461	39	0.95 (0.93, 0.97)	0.89 (0.86, 0.93)	0.89 (0.85, 0.93)	
Age, years:						0.838
0: <13	326	27	0.95 (0.92, 0.98)	0.9 (0.86, 0.94)	0.89 (0.85, 0.94)	
1: ≥13	135	12	0.96 (0.92, 1)	0.88 (0.81, 0.95)	0.88 (0.81, 0.95)	
Gender:						0.923
Female	200	16	0.94 (0.9, 0.98)	0.89 (0.83, 0.95)	0.89 (0.83, 0.95)	
Male	261	23	0.96 (0.93, 0.99)	0.9 (0.85, 0.95)	0.89 (0.84, 0.94)	
Race/Ethnicity:						0.702
Asian	31	2	1 (1, 1)	0.94 (0.84, 1)	0.94 (0.84, 1)	
Black/African American	46	7	0.91 (0.82, 1)	0.85 (0.73, 0.98)	0.85 (0.73, 0.98)	
Hispanic or Latino	182	17	0.94 (0.9, 0.98)	0.88 (0.82, 0.94)	0.86 (0.8, 0.93)	
Other	2	0	1 (1, 1)	1 (1, 1)	1 (1, 1)	
White	162	11	0.96 (0.92, 1)	0.92 (0.86, 0.97)	0.92 (0.86, 0.97)	
PBT with Anesthesia:						0.558
None	267	20	0.96 (0.93, 0.99)	0.9 (0.86, 0.95)	0.89 (0.84, 0.94)	
Propofol Anesthesia	194	19	0.94 (0.9, 0.98)	0.88 (0.83, 0.94)	0.88 (0.83, 0.94)	
Prior Chemotherapy:						0.152
No	379	28	0.96 (0.93, 0.98)	0.91 (0.87, 0.95)	0.9 (0.86, 0.94)	
Yes	82	11	0.92 (0.86, 0.99)	0.84 (0.75, 0.95)	0.84 (0.75, 0.95)	
Concurrent Chemotherapy:						0.003
No	397	27	0.96 (0.94, 0.99)	0.92 (0.88, 0.95)	0.91 (0.87, 0.95)	
Yes	64	12	0.88 (0.8, 0.98)	0.79 (0.68, 0.92)	0.79 (0.68, 0.92)	
PBT Interrupted:						0.019
No	232	14	0.98 (0.96, 1)	0.93 (0.89, 0.97)	0.92 (0.87, 0.97)	
Yes	229	25	0.92 (0.88, 0.96)	0.86 (0.8, 0.92)	0.86 (0.8, 0.92)	
Number of Interruptions:						0.033
0 days	232	14	0.98 (0.96, 1)	0.93 (0.89, 0.97)	0.92 (0.87, 0.97)	
1 day	93	8	0.95 (0.9, 1)	0.91 (0.83, 0.99)	0.91 (0.83, 0.99)	
≥2 days	136	17	0.80 (0.84, 0.96)	0.83 (0.75, 0.91)	0.83 (0.75, 0.91)	

Abbreviations: OS, overall survival: NA, not attained: PBT, proton beam therapy. * *p*-value from the log-rank test.

**Table 3:** Patient characteristics stratified by unplanned admissions or emergency room visits within 30 days of the start of proton beam therapy.

Variable	30-Day Unplanned Admission and/ or Emergency Room Visit (Before Propensity Score Matching)				30-Day Unplanned Admission and/or Emergency Room Visit (After Propensity Score Matching)			
	All (*n* = 461)	No (*n* = 434)	Yes (*n* = 27)	*p*-Value	All (*n* = 296)	No (*n* = 278)	Yes (*n* = 18)	*p*-Value
Age, years (median, IQR)	9 (5, 13)	9 (5, 13)	4 (2, 8)	<0.001	8.0 (4, 12)	8.0 (4.0, 13.0)	6.0 (4.0, 8.0)	0.099
Gender, *n* (%)				0.278				
Female	200 (43.4)	191 (95.5)	9 (4.5)					
Male	261 (56.6)	243 (93.1)	18 (6.9)					
Ethnicity/Race, *n* (%)	2 (0.5)	2 (100)	0 (0)	0.725				
AIAN	31 (7.3)	29 (93.5)	2 (6.5)					
Asian	46 (10.9)	44 (95.7)	2 (4.3)					
Black/AA								
Hispanic or Latino	182 (43)	169 (92.9)	13 (7.1)					
White	162 (38.3)	155 (95.7)	7 (4.3)					
PBT with Propofol Anesthesia, *n* (%)				<0.001				<0.001
No	267 (57.9)	266 (99.6)	1 (0.4)		148 (50.0)	147 (99.3)	1 (0.7)	
Yes	194 (42.1)	168 (86.6)	26 (13.4)		148 (50.0)	131 (88.5)	17 (11.5)	
Previous Chemotherapy, *n* (%)				0.029				0.1427
No	379 (82.2)	361 (95.3)	18 (4.7)		260 (87.8)	242 (93.1)	18 (6.9)	
Yes	82 (17.8)	73 (89)	9 (11)		36 (12.2)	36 (100.0)	0 (0.0)	
Concurrent Chemotherapy				0.403				
No	397 (86.1)	375 (94.5)	22 (5.5)					
Yes	64 (13.9)	59 (92.2)	5 (7.8)					
Number of Chemotherapy Agents, *n* (%)				0.101				
None	346 (75.1)	330 (95.4)	16 (4.6)					
One	31 (6.7)	27 (87.1)	4 (12.9)					
Two	34 (7.4)	32 (94.1)	2 (5.9)					
Three or more	50 (10.8)	45 (90)	5 (10)					
Days between Cancer-Related Surgery and Start of PBT, mean (SD)	241 (569)	245 (578)	164(402)	0.834				
Proton Radiotherapy Field, No. (%)				0.182				0.1302
Brain	234 (50.8)	222 (94.9)	12 (5.1)		122 (41.2)	114 (93.4)	8 (6.6)	
Craniospinal	207 (44.9)	195 (94.2)	12 (5.8)		164 (55.4)	156 (95.1)	8 (4.9)	
Spine	20 (4.3)	17 (85.0)	3 (15)		10 (3.4)	8 (80.0)	2 (20.0)	
PBT Interrupted, *n* (%)				0.027				
No	232 (50.3)	224 (96.6)	8 (3.4)					
Yes	229 (49.7)	210 (91.7)	19 (8.3)					
Days of Interruption, *n* (%)				0.013				
1	93 (20.2)	89 (95.7)	4 (4.3)					
≥2	136 (29.5)	121 (89)	15 (11)					

Abbreviations: IQR, interquartile range; AIAN, American Indian or Alaska Native; AA, African American; PBT, proton beam therapy.

## Data Availability

The datasets presented in this article are not readily available because of our privacy policy.

## Supplementary Materials

The following supporting information can be downloaded at https://www.mdpi.com/article/10.3390/cancers17121904/s1, Table S1: Assessment of Covariate Distribution after Propensity Score Matching; Table S2: Symptoms at 30-Day Unexpected Readmission or Emergency Room Visit in 461 Children Undergoing Proton Beam Therapy; Table S3. Diagnoses at Unexpected Readmission or Emergency Room Visit.
