# Supplementary material for: Propofol Total Intravenous Anesthesia for Pediatric Proton Radiotherapy and Its Effect on Patient Outcomes
Source: Cancers (Basel). 2025 Jun 7;17(12):1904. doi: 10.3390/cancers17121904 (PMC12191059; doi:10.3390/cancers17121904)
Supplement: Supplementary file 1 [file cancers-17-01904-s001.zip › cancers-3527133-supplementary.pdf]

## Supplemental Tables

**Supplemental Table S1.** Assessment of Covariate Distribution after Propensity Score Matching

| Variable | Proton Radiotherapy with Propofol Anesthesia | | Total |
|---|---|---|---|
| | No (N = 148) | Yes (N = 148) | (N = 296) |
| Chemotherapy History, n (%) | | | |
| No | 130 (87.8%) | 130 (87.8%) | 260 (87.8%) |
| Yes | 18 (12.2%) | 18 (12.2%) | 36 (12.2%) |
| Proton Radiotherapy Field, n (%) | | | |
| Brain | 61 (41.2%) | 61 (41.2%) | 122 (41.2%) |
| Craniospinal | 82 (55.4%) | 82 (55.4%) | 164 (55.4%) |
| Spine | 5 (3.4%) | 5 (3.4%) | 10 (3.4%) |

**Supplemental Table S2.** Symptoms at 30-Day Unexpected Readmission or Emergency Room Visit in 461 Children Undergoing Proton Beam Therapy.

| Variable | Levels | No Anesthesia | Propofol Anesthesia | All Patients | *p*-Value |
|---|---|---|---|---|---|
| 30-day Admission and/or ER visit, n (%) | Yes<br>No | 1 (0.4)<br>266 (99.6) | 26 (13.4)<br>168 (86.6) | 27 (5.9)<br>434 (94.1) | < 0.001 |
| Symptoms at Readmission or ER Visit | | | | | |
| Pain, n (%) | Yes<br>No | 1 (0.4)<br>266 (99.6) | 1 (0.5)<br>193 (99.5) | 2 (0.4)<br>459 (99.6) | 1.000 |
| Neurologic, n (%) | Yes<br>No | 0 (0)<br>267 (100) | 8 (4.1)<br>186 (95.9) | 8 (1.7)<br>453 (98.3) | < 0.001 |
| Pulmonary, n (%) | Yes<br>No | 0 (0)<br>267 (100) | 2 (1)<br>192 (99) | 2 (0.4)<br>459 (99.6) | 0.177 |
| Gastrointestinal, n (%) | Yes<br>No | 0 (0)<br>267 (100) | 2 (1)<br>192 (99) | 2 (0.4)<br>459 (99.6) | 0.177 |
| Bleeding, n (%) | Yes<br>No | 0 (0)<br>267 (100) | 1 (0.5)<br>193 (99.5) | 1 (0.2)<br>460 (99.8) | 0.421 |
| Nausea and Vomiting, n (%) | Yes<br>No | 0 (0)<br>267(100) | 5 (2.6)<br>189 (97.4) | 5 (1.1)<br>456 (98.9) | 0.013 |
| Fever, n (%) | Yes<br>No | 0 (0)<br>267 (100) | 8 (4.1)<br>186 (95.9) | 8 (1.7)<br>453 (98.3) | < 0.001 |
| Infection, n (%) | Yes<br>No | 0 (0)<br>267 (100) | 5 (2.6)<br>189 (97.4) | 5 (1.1)<br>456 (98.9) | 0.013 |
| Other, n (%) | Yes<br>No | 0 (0)<br>267 (100) | 2 (1)<br>192 (99) | 2 (0.4)<br>459 (99.6) | 0.177 |

Abbreviations: ER, emergency room.

**Supplemental Table S3.** Diagnoses at Unexpected Readmission or Emergency Room Visit

| | **No-Anesthesia (n = 267)** | **Propofol-Anesthesia (n = 194)** |
|---|---|---|
| All Diagnoses and symptoms, n (%) | 1 (0.4) | 27 (14.0) |
| All Infectious, n (%): | 0 (0) | 13 (6.7) |
| Bacteremia | | 1 (0.5) |
| CVC site erythema | | 1 (0.5) |
| Fever | | 2 (1.0) |
| Headache, vomiting fever | | 1 (0.5) |
| Nausea, vomiting, fever | | 1 (0.5) |
| Pneumonia | | 2 (1.0) |
| Sepsis | | 2 (1.0) |
| Urinary tract infection | | 1 (0.5) |
| Viral gastroenteritis | | 1 (0.5) |
| Wound infection | | 1 (0.5) |
| All Gastrointestinal, n (%) | 0 (0) | 2 (1.0) |
| Constipation | | 1 (0.5) |
| Diarrhea | | 1 (0.5) |
| All Neurologic, n (%) | 1 (0.4) | 7 (3.6) |
| Cerebrovascular accident | | 1 (0.5) |
| Dizziness | | 1 (0.5) |
| Esotropia | | 1 (0.5) |
| Headaches | 1 (0.4) | 0 (0) |
| Hemiparesis | | 2 (1.0) |
| Non-traumatic brain injury | | 1 (0.5) |
| Paraplegia | | 1 (0.5) |
| Other Diagnoses and Symptoms, n (%) | 0 (0) | 5 (2.6) |
| CVC cut and bleeding | | 1 (0.5) |
| Fatigue | | 1 (0.5) |
| Dyspnea | | 2 (1.0) |
| Hemoptysis | | 1 (0.5) |

Abbreviations: CVC, central venous catheter
